# Expanding outpatient benefits package can reduce diabetes-related avoidable hospitalizations

**DOI:** 10.3389/fpubh.2023.964789

**Published:** 2023-02-14

**Authors:** Hao-Ran Liu, Si-Yuan Chen, Lan-Yue Zhang, Hong-Qiao Fu, Wei-Yan Jian

**Affiliations:** Department of Health Policy and Management, School of Public Health, Peking University Health Science Center, Beijing, China

**Keywords:** diabetes mellitus, avoidable hospitalizations, outpatient benefits package, substitution effect of outpatient services, China

## Abstract

**Objective:**

To evaluate the policy effect of replacing hospitalization service with outpatient service and reducing diabetes-related avoidable hospitalizations by improving outpatient benefits package.

**Methods:**

A database of hospital discharge from 2015 to 2017 in City Z was used. All diabetic inpatient cases enrolled in Urban Employee Basic Medical Insurance were selected as the intervention group, and diabetic inpatient cases enrolled in Urban and Rural Resident Basic Medical Insurance were selected as the control group. The Difference-in-Difference model was used to analyze the effect of improving outpatient benefits package level of diabetes from 1800 yuan (about $252.82) to 2400 yuan (about $337.09) per capita per year on avoidable hospitalization rate, average hospitalization cost and average length of stay.

**Results:**

The avoidable hospitalization rate of diabetes mellitus decreased by 0.21 percentage points (*P* < 0.01), the average total cost of hospitalization increased by 7.89% (*P* < 0.01), and the average length of stay per hospitalization increased by 5.63% (*P* < 0.01).

**Conclusions:**

Improving the outpatient benefits package of diabetes can play a role in replacing hospitalization service with outpatient service, reducing diabetes-related avoidable hospitalizations, and reducing the disease burden and financial burden.

## 1. Introduction

Diabetes mellitus is one of the leading non-communicable diseases causing death in people aged 30 to 70 years worldwide (1). Diabetes and its complications have been imposing substantial financial burden of health systems and disease burden for patients and their families. According to the 10th edition of the IDF Diabetes Atlas 2021, the estimated global direct health expenditure on diabetes is $966 billion in 2021 and could reach $1.05 trillion by 2045 (2). Reducing the burden of diabetes is a common goal for all countries around the world. China has the largest number of diabetic patients in the world (2). The total number of adults with diabetes in mainland China is estimated to be 140.9 million, with an estimated 174.4 million in 2045 and an adult prevalence of 13.0 % (2). In 2021, diabetes-related health costs in China are as high as $165.3 billion, and the prevalence of diabetes in China continues to grow (2–5).

Avoidable hospitalizations are those that can be avoided through timely, effective, and adequate primary health care services (6). If patients have access to timely and effective ambulatory care, it is possible to reduce hospitalizations for these conditions by preventing the occurrence of diseases or managing chronic conditions in an outpatient setting (7). Diabetes

mellitus has now been included in many index systems of avoidable hospitalizations diseases across the world (8–11). If the incidence of avoidable diabetes hospitalizations and their associated complications can be reduced, it will not only save medical costs, but also help improve the quality of primary health care service and slow down the disease process, thus reducing the disease burden of diabetes.

In many countries, great efforts have been made to reduce diabetes-related avoidable hospitalizations (DRAHs) through the substitution effect of outpatient services. There are three main mechanisms to replace inpatient services with primary care (12). First, increasing outpatient services to avoid the need for inpatient treatment. For example, Hungary has increased outpatient costs to reduce inpatient costs (13). Second, managing the health conditions with chronic diseases, such as the teach-back methods for outpatients in the United States (14), home healthcare within 14 days after hospital discharges (15), and the patient-centered Care Coordination Home Telehealth (16). Third, utilizing the role of general practitioners (GP) as the gatekeepers to reduce referrals, such as Italy increasing the income of GPs in outpatient clinics to reduce the referral rate of outpatients (17), and the United States strengthening the level of communication between primary care and specialists physicians in outpatient visits (18). All these measures are beneficial in reducing DRAHs.

In mainland China, the concept of avoidable hospitalization was introduced late and has attracted few attentions in the last 5 years. Most of the current literature focuses on the measurement of avoidable hospitalizations (19–22), with little assessment of the impact of existing policies on avoidable hospitalization. Therefore, using data from City Z (which we refer to “City Z” because the data provider does not want to disclose the name of the city), we empirically studied the impact of improved the outpatient benefits package on DRAHs. As we used the natural experiment in City Z and employed the Difference-in-Difference (DID) model, we could observe the causality between outpatient benefits package expansion and avoidable hospitalization.

In the City Z, the maximum annual payment limit of outpatient benefits package for diabetes who enrolled in Urban Employee Basic Medical Insurance (UEBMI) has been raised from 1,800 yuan (about $252.82) to 2,400 yuan (about $337.09) since January 1, 2016. If patients can take full advantages of health-care services to delay the progression of diabetes, then diabetes hospitalizations can be reduced. Therefore, our study aims to analyze the effect of improving outpatient reimbursement level from 1,800 yuan (about $252.82) to 2,400 yuan (about $337.09) per capita per year on DRAHs. In the context of today's lack of response to avoidable hospitalizations, evaluating the outpatient substitution effect and determining whether better health care provided by outpatient services can reduce avoidable hospitalizations will benefit not only China, but also other low-and middle-income countries (LMICs).

## 2. Material and methods

### 2.1. Study design

Since 2007, City Z has implemented a medical insurance policy to include diabetes in the priority treatment of outpatient chronic disease policy. The medical insurance fund specifies the maximum amount of payment for specialized medicines in the outpatient service of patients. The reimbursement level of basic medical insurance for diabetic inpatients who enrolled in UEBMI is 1,800 yuan (about $252.82) per person per year, while that for diabetic inpatients who enrolled in Urban and Rural Resident Basic Medical Insurance (URRBMI) is 1,200 yuan (about $168.55) per person per year. Since January 1, 2016, the level of reimbursement has changed with the implementation of the new policy. The outpatient benefits package for urban employee with diabetes increased by 600 yuan (about $84.28) per year, while that for urban and rural residents remained unchanged at 1,200 yuan (about $168.55) per year. Therefore, in order to evaluate the impact of expanded outpatient benefits package on DRAHs, we chose diabetic inpatients who enrolled in UEBMI as the intervention group and diabetic inpatients who enrolled in URRBMI as the control group. We use the DID model to examine the impact of improving outpatient benefits package level of diabetes from 1,800 yuan (about $252.82) to 2,400 yuan (about $337.09) per capita per year on DRAHs.

### 2.2. Data sources

In this study, the database of inpatient discharges from 2015 to 2017 was obtained from the health administration department of city Z, a developed city in eastern China. The data included patients' basic information, hospitalization information, and cost information. Basic information included patient ID, current address, age, gender, marital status, and nationality. Hospitalization information includes admission time, discharge time, visit time, length of stays, primary diagnosis and International Classification of Diseases (ICD) code, secondary diagnosis and ICD code, primary surgical operation and ICD code, and secondary surgical operation and ICD code. Cost information includes total hospitalization expenses, out-of-pocket hospitalization expenses, drug expenses, and consumable expenses.

The database contains discharge data of inpatients from all medical institutions in the city. After screening the avoidable hospitalizations and addressing the outliers and missing values, all data were included in the analysis and patient information has been desensitized. Comparable price adjustments were made to the relevant cost data using the China Consumer Price Index. The definition of avoidable hospitalization from “Health Care Quality and Outcomes (HCQO) 2020–2021 Indicator Definitions” was published by the Organization for Economic Co-operation and Development (OECD) in 2020. The inclusion and exclusion criteria for DRAHs were defined in detail by OECD (10). OECD indicators provide guidance for comparing the performance of health systems among member countries, making avoidable hospitalizations comparable across countries and facilitating cross-country comparisons of avoidable hospitalizations (23, 24). The single DRAHs indicator of the OECD is created by combining three widely used avoidable hospitalization indicators for people with diabetes as follows: uncontrolled diabetes without complications, diabetes with short-term complications, and diabetes with long-term complications. The specific inclusion and exclusion criteria are as follows.

Inclusion criteria: (1) age ≥15 years. All acute care hospitals patients, including public and private hospitals that provide inpatient care. (2) The primary discharge diagnosis was coded as diabetes. The first three ICD-10 codes were “E10”, “E11”, “E13” and “E14”.

Exclusion criteria: (1) Cases where the patient died in hospital during the admission. (2) Cases resulting from a transfer from another acute care institution (transfers-in). (3) Cases with MDC 14 or specified pregnancy, childbirth, and puerperium codes in any field. (4) Cases that are same day/day only admissions.

### 2.3. Indicators

The primary variables in this study were avoidable hospitalization rate, average hospitalization cost and average length of stay. Of all hospitalized patients during the year, those who belongs to avoided hospitalization were assigned a value of 1; otherwise, it equaled 0. The total cost per hospitalization and the average length of stay were transformed by logarithm.

The study controlled for the effects of three variables. We included age (15–40, 41–60, 61–80, >80), gender (female, male), and CCI (0, 1, ≥2). In order to analyze the basic information of avoidable hospitalization cases, we used the Charlson Comorbidity Index (CCI), which evaluates the severity of the damage and the abnormalities of the patient's other organs or tissues other than the underlying disease, allowing the patients to be classified according to the severity of comorbidities. The CCI is based on the ICD-10 and its calculation based on all secondary diagnoses of the patients. The higher the score, the more severe the disease comorbidity. CCI was divided into three groups (25, 26). Age was grouped according to the quintile method. Because the inclusion criteria for the population were age ≥15 years, age was divided into four groups.

To validate the increase in the average severity of hospitalized patients with diabetes after policy changes, we defined the CCI group variable. CCI group =0 if CCI =0 or 1 and CCI group =1 if CCI ≥2.

### 2.4. Statistical analysis

The DID model was used to analyze the effect of improving outpatient benefit package level of diabetes from 1,800 yuan (about $252.82) to 2,400 yuan (about $337.09) per capita per year on avoidable hospitalization rate, average total cost per hospitalization and average length of stay. The model is as follows:


$$\begin{array}{l} Y_{i}=\beta_{0}+\alpha {\;*\;T1+\beta }_{1}\;*\;Reform_{i}+\beta_{2}\;*\;Intervention_{i}\\ \;+\beta_{3}\;*\;Reform_{i}\;*\;Intervention_{i}+\delta \;*\;T1\;*\;Reform_{i}+\underset{j}{\sum }\delta_{j}x_{ji}\\ \;+\varepsilon_{ih} \end{array}$$

The avoidable hospitalization rate in this model was defined as the proportion of avoidable hospitalizations in all hospitalizations during the current year, thus, in the regression model, hospitalization cases were represented by 1 and other cases were by 0. The two models of average total cost and the average length of stay corresponded to the average total cost, and the average length of stay after the logarithmic conversion, respectively. *Reform*_*i*_ was a dummy variable of time points before and after the implementation of the policy. *Reform*_*i*_=1 if year = 2016 and later, *Reform*_*i*_=0 if year = 2015. β_1_ reflected the difference before and after the implementation of the policy. *Intervention*_*i*_ was a dummy variable for grouping, where the intervention group was 1 and the control group was 0. β_2_ reflected the difference between the intervention group and the control group. The coefficient β_3_ of the interaction term $Reform_{i}^{*}Intervention_{i}$ estimated by the model was the actual impact of the policy. *x*_*ji*_ was a confounding factor that needs to be controlled, including age, gender, and CCI. ε_*ih*_ was the residual value. A parallel trend test was conducted by using data from the year of 2015 to test whether the trends in the intervention group and the control group were parallel before the policy change. *T*1 = 0 when year = 2015 and the month = from January to April. *T*1 = 1 when year = 2015 and the month = from May to December. If δ = 0, the parallel trend hypothesis of the control group and the intervention group before the policy change is established. The coefficient *β*_3_ of the interaction term $Reform_{i}^{*}Intervention_{i}$ reflected the actual impact of the policy. If δ ≠ 0, the net effect of the policy is *β*_3_ minus *δ*. The DID model was also performed on the CCI group to verify whether policy changes could increase the average severity of diabetic inpatients. The robust test was used in parameter estimation.

All data analysis was conducted using Stata 16.0 software.

## 3. Results

### 3.1. Descriptive data

According to the inclusion and exclusion criteria, a total of 73,750 cases of avoidable hospitalization were included from 2015 to 2017. There were 55,528 cases in the intervention group and 18,222 cases in the control group. We described the baseline of avoidable hospitalizations before and after the policy change (2015 before the change, 2016–2017 after the change) in the control and intervention groups by age, sex and CCI (Table 1). The 61–80 age group had the highest number of people, while the 15–40 age group had the lowest. Both before and after the policy change, the control group had slightly more females than males, while the intervention group had slightly more males than females. The distribution of CCI in the intervention group and the control group was approximately the same, with the highest proportion in the group 0 (about 50%), and a similar number in group 1 as in group 2 and above, the proportion in group 2 and above in the intervention group was slightly higher than that in the control group.

**Table 1:** Basic information of avoidable hospitalization cases in the intervention and control groups before and after the policy change.

		**Before the policy**		**After the policy**	
	**Control group**	**Intervention group**	**Control group**	**Intervention group**
*N =*	4,113	14,735	14,109	40,793
**Age**				
15–40 (*N*, %)	280 (6.801)	854 (5.796)	839 (5.947)	2493 (6.111)
41–60 (*N*, %)	1,530 (37.20)	5,368 (36.43)	4,316 (30.59)	13,198 (32.35)
61–80 (*N*, %)	2,074 (50.43)	6,859 (46.55)	7,455 (52.84)	18,659 (45.74)
>80 (*N*, %)	229 (5.568)	1,654 (11.22)	1,499 (10.62)	6,443 (15.79)
**Gender**				
Female (*N*, %)	2,168 (52.71)	7,265 (49.30)	7,171 (50.83)	19,825 (48.60)
Male (*N*, %)	1,945 (47.29)	7,470 (50.70)	6,938 (49.17)	20,968 (51.40)
**CCI**				
0 (*N*, %)	2,107 (51.23)	7,292 (49.49)	7,195 (51.00)	19,741 (48.39)
1 (*N*, %)	1,137 (27.64)	4,093 (27.78)	3,800 (26.93)	10,780 (26.43)
≥2 (*N*, %)	869 (21.13)	3,350 (22.73)	3,114 (22.07)	10,272 (25.18)

### 3.2. Change of trends

Table 2 showed the absolute changes in the avoidable hospitalization rate, average hospitalization cost per admission, the length of stay, age, gender and CCI for both intervention and control groups before and after the policy change. Compared with the control group, the avoidable hospitalization rate decreased in intervention group, while the average hospitalization cost and the absolute length of stay per hospitalization increased after the policy. Specifically, the total rate of avoidable hospitalizations decreased by 0.109%, the average hospitalization cost increased by 495 yuan (about $69.53), and the average hospitalization duration increased by 0.48 days. Compared with the control group, the group 2 and above of CCI increased in the intervention group, while the proportion of the group 0 and group 1 decreased after the policy implementation. Specifically, the percentage of patients in group 0 decreased by 0.87%, while the percentage in group 1 decreased by 0.64%, and the percentage in group 2 and above increased by 1.51%.

**Table 2:** Absolute changes in avoidable hospitalization rate, costs, length of hospitalization, age, gender and CCI in the intervention and control groups before and after the policy.

**Indicators**	**Before the policy**			**After the policy**			**Difference-in-difference**
	**Intervention group**	**Control group**	**Difference**	**Intervention group**	**Control group**	**Difference**	
Avoidable hospitalization rate (%)	2.512	1.791	0.721	2.72	2.108	0.612	−0.109
Average hospitalization cost (yuan)	11,226 ($1,576.75)	9,912 ($1,392.20)	1,314 ($184.55)	11,004 ($1,545.57)	9,195 ($1,291.49)	1,809 ($254.08)	495 ($69.53)
Average hospitalization duration (day)	10.39	9.841	0.549	10.48	9.451	1.029	0.48
**Age**							
15–40 (%)	5.796	6.801	−1.005	6.111	5.947	0.164	1.169
41–60 (%)	36.43	37.20	−0.77	32.35	30.59	1.76	2.53
61–80 (%)	46.55	50.43	−3.88	45.74	52.84	−7.10	−3.22
>80 (%)	11.22	5.568	5.652	15.79	10.62	5.17	−0.482
**Gender**							
Female (%)	49.30	52.71	−3.41	48.60	50.83	−2.23	1.18
Male (%)	50.70	47.29	3.41	51.40	49.17	2.23	−1.18
**CCI**							
0 (%)	49.49	51.23	−1.74	48.39	51.00	−2.61	−0.87
1 (%)	27.78	27.64	0.14	26.43	26.93	−0.50	−0.64
≥2 (%)	22.73	21.13	1.60	25.18	22.07	3.11	1.51

### 3.3. Policy effects

The DID model shown in Table 3 was applied to analyze the impact of the medical insurance policy reform on diabetes. Since 2016, the increase in medical insurance reimbursement had a significant effect on the avoidable hospitalization rate, the average hospitalization cost, and the average length of hospitalization. The avoidable hospitalization rate of diabetes mellitus decreased by 0.21 percentage points (*P* < 0.01), the total cost of hospitalization increased by 7.89% (*P* < 0.01), and the length of hospitalization increased by 5.63% (*P* < 0.01), with all the differences statistically significant. The CCI group of diabetes mellitus increased by 2.21% (*P* < 0.01). As the avoidable hospitalization rate was ~2.5% in the intervention group during the study period, the decrease of 0.21 percentage points in avoidable hospitalization rate due to expansion of outpatient benefits package suggested that the volume of avoidable hospitalization fell by 8.4% (=0.21/2.5).

**Table 3:** Effects of policy change on avoidable hospitalization for diabetes mellitus (DID model).

**Indicators**		**Avoidable hospitalization rate**	**Average hospitalization cost**	**Average hospitalization duration**	**CCI group**
δ		−0.0007	0.0125	0.0214	0.0083
Net effect of policy		−0.0021^***^ (0.000)	0.0789^***^ (0.013)	0.0563^***^ (0.012)	0.0221^***^ (0.008)
Before and after the policy		0.0044^***^ (0.000)	−0.0829^***^ (0.011)	−0.0592^***^ (0.011)	−0.0065 (0.007)
Intervention group		0.0037^***^ (0.000)	0.1392^***^ (0.011)	0.0812^***^ (0.010)	0.0063 (0.007)
Age	15–40	0.0072^***^ (0.000)			
	41–60	0.0287^***^ (0.000)	0.1053^***^ (0.010)	0.0634^***^ (0.010)	0.1021^***^ (0.004)
	61–80	0.0286^***^ (0.000)	0.1796^***^ (0.010)	0.1691^***^ (0.010)	0.2345^***^ (0.004)
	>80	0.0195^***^ (0.000)	0.2255^***^ (0.011)	0.2864^***^ (0.011)	0.3192^***^ (0.006)
Gender (Female = Reference group)	Male	0.0044^***^ (0.000)	−0.0052 (0.004)	−0.0129^***^ (0.004)	0.0473^***^ (0.003)
CCI (0= Reference group)	1	0.0194^***^ (0.000)	0.1219^***^ (0.005)	0.1098^***^ (0.005)	
	≥2	0.0079^***^ (0.000)	0.2431^***^ (0.006)	0.1289^***^ (0.005)
Constant		−0.0073^***^ (0.000)	8.7372^***^ (0.014)	1.9179^***^ (0.013)	0.0149^**^(0.007)
*N =*		2,985,434	73,748	73,750	73,750
R-squared		0.009	0.067	0.048	0.044

The data of hospitalization cost and length of stay were analyzed by logistic regression analysis, and the robust standard error was found in parentheses. Age, sex, and CCI were all controlled by the model.

*** P < 0.01,

** P < 0.05,

^*^ P < 0.1.

The parallel trend test results are shown in Table 4. We suppose the policy changes happened on May 1, 2015 and used the data in 2015 to conduct the DID analysis. The results show that the coefficient on variables of interest are all statistically insignificant, suggesting that the heterogeneous trends in the intervention group and the control group before the policy change could not drive the findings in our study.

**Table 4:** Parallel trends test before policy change (DID model).

**Indicators**		**Avoidable hospitalization rate**	**Average hospitalization cost**	**Average hospitalization duration**
Effect of policy		−0.0007 (0.001)	0.0125 (0.024)	0.0214 (0.023)
Before and after the policy		0.0004 (0.001)	−0.0322 (0.022)	−0.0243 (0.021)
Intervention group		0.0046^***^ (0.001)	0.1297^***^ (0.020)	0.0658^***^ (0.019)
Age	15–40	0.0061^***^ (0.000)		
	41–60	0.0273^***^ (0.000)	0.0961^***^ (0.021)	0.0190 (0.020)
	61–80	0.0247^***^ (0.000)	0.1605^***^ (0.021)	0.1181^***^ (0.0020)
	>80	0.0116^***^ (0.000)	0.2278^***^ (0.026)	0.2533^***^ (0.023)
Gender (Female=Reference group)	Male	0.0028^***^ (0.000)	−0.0106 (0.009)	−0.0089 (0.008)
CCI (0= Reference group)	1	0.0235^***^ (0.001)	0.1241^***^ (0.011)	0.1214^***^ (0.010)
	≥2	0.0048^***^ (0.000)	0.2348^***^ (0.012)	0.1152^***^ (0.011)
Constant		−0.0043^***^ (0.000)	8.7762^***^ (0.027)	1.9768^***^ (0.026)
*N =*		816,287	18,848	18,848
R-squared		0.009	0.048	0.039

The data of hospitalization cost and length of stay were analyzed by logistic regression analysis, and the robust standard error was found in parentheses. Age, sex, and CCI were all controlled by the model.

*** P < 0.01,

^**^ P < 0.05, ^*^ P < 0.1.

## 4. Discussion

The study found that the avoidable hospitalization rate of diabetes mellitus decreased, the total cost of hospitalization and the length of hospitalization increased after the new policy was implemented in city Z. There are some similar studies. Following the introduction of financial incentives for the Emilia-Romagna in Italy, Elisa Iezzi et al. used a model to measure the impact of different levels of incentives for GPs on DRAHs in each region (17). The model showed that for every 100 euros (~17% of the annual income of the GP diabetes program) increase in financial incentives paid to GPs, there was an average 1% reduction in avoidable diabetes hospitalizations and an average reduction of ~100 cases across the region. Meanwhile, uninsured or underinsured groups had a higher rate of avoidable hospitalizations than the insured groups (27). After the implementation of a health transformation program in a hospital in western Iran, the average cost of hospitalization for diabetes increased from $372.55 to $1,119.77, and the average length of stay increased from 5.6 days to 7.57 days (28). Tables 2, 3 showed that compared with control group, the group 2 and above of CCI increased in the intervention group, while the proportion of the group 0 and group 1 decreased after the policy implementation. There was an increased proportion of avoidable diabetes hospitalizations with severe comorbidities.

This study found that expanding outpatient benefits package for diabetes can contribute to reduce avoidable hospitalizations, which is consistent with the results of Meng-Han Shen (29), Yang Fan (30) and Feng-Mei Zhu (31), which concluded that outpatient services have a substitution effect on inpatient services (13, 32–35). Previous studies have found that increasing the motivation of primary health care providers and strengthening the management of patients with chronic diseases can help reduce avoidable hospitalizations (17). The increased use of outpatient services and outpatient reimbursement insurance can significantly reduce the cost of inpatient services in China's new rural cooperative medical system (34). Poor outpatient benefits package may bring some adverse impacts. First, patients cannot receive the expected utilization of outpatient service and will have a heavy burden of outpatient medical costs. Thus, patients prefer to reduce the utilization of medical services and the usage of inpatient services may increase by the disease progression. Second, some patients with mild illness are dissatisfied with the outpatient benefits package so they prefer hospitalization treatment to receive higher reimbursement and alleviate the burden of personal expenses. As the outpatient benefits package expands, patients have an increased incentive to use outpatient services, thereby controlling the disease at an early stage and reducing hospitalization (31). If the substitution effect of outpatient service can be exerted, the average hospitalization cost and average length of stay of inpatients will increase with the decrease of avoidable hospitalization rate. This is due to an increase in the average severity of diabetes inpatients following a reduction in avoidable hospitalizations.

This study was based on China, one of the LMICs, to examine the impact of policy effects on avoidable hospitalization. First, we describe the absolute changes in avoidable hospitalizations before and after policy changes. Second, our results show that expanding the outpatient benefits package for diabetes can reduce DRAHs, and explain its underlying causes. This has implications for the further improvement of primary health care in LMICs. At the beginning of the establishment of medical insurance system in China, the main purpose was to guarantee hospitalization service. The intention of the medical insurance system was to help the insured cope with the high risk of disease and improve the efficiency of the use of medical insurance funds in the face of limited financing capacity (36–38). However, the side-effect of the operation of the system is the overutilization of inpatient services, which undermines the efficiency of the utilization of the medical insurance fund (38–40). The policy change of city Z deserves further replication. Furthermore, in the light of international experience, there is a need for in-depth reform to improve the quality of primary healthcare services, including management of diabetic patients and improve the effectiveness of disease prevention and control. DRAHs can further be reduced.

Our study has some limitations. First, we were not able to analyze the influence of the policy on the outpatient service utilization because of the lack of the outpatient service utilization data in city Z. Second, expanding the outpatient benefits package leads to higher costs of primary care, while less DRAHs leads to lower costs for in-patients. This study did not further examine the overall economic benefits of policy. Third, the policy was implemented on January 1, 2016. The study only included data for 2 years after the policy change and did not observe long-term effects of policy implementation.

## 5. Conclusion

Expanding the outpatient benefits package for diabetes can reduce avoidable hospitalizations, and allow limited resources for inpatient services to be reserved for the treatment of serious cases that requires inpatient care, which assists in improving the overall efficiency of the health system. More importantly, patients would benefit from more timely and effective diagnosis and treatment, rather than delayed treatment. LMICs, including China, need to invest more in primary healthcare services and increase the capacity of primary healthcare services to make the overall health system more organized and efficient.

## Data availability statement

The raw data supporting the conclusions of this article will be made available by the authors, without undue reservation.

## Author contributions

W-YJ and H-QF led the design of the study and contributed to the interpretation of the results. H-RL conducted statistical analysis and drafted the manuscript. S-YC participated in paper writing and statistical analysis. L-YZ participated in paper writing. H-QF organized the database. All authors have read and approved the final draft.

## References

[1] WHO. World Health Statistics 2021: Monitoring health for the SDGs. (2021). Geneva: WHO

[2] International Diabetes Federation. IDF Diabetes Atlas, 10th ed. Brussels, Belgium: International Diabetes Federation (2021). Available online at: https://www.diabetesatlas.org.

[3] Wang L Peng W Zhao Z Zhang M Shi Z Song Z. Prevalence and Treatment of Diabetes in China, 2013-2018. JAMA. (2021) 326:2498–506. 10.1001/jama.2021.22208 34962526 PMC8715349

[4] Williams R Karuranga S Malanda B Saeedi P Basit A Besançon S. Global and regional estimates and projections of diabetes-related health expenditure: Results from the International Diabetes Federation Diabetes Atlas, 9th edition. Diabetes Res Clin Pract. (2020) 162:108072. 10.1016/j.diabres.2020.108072 32061820

[5] Yang W Lu J Weng J Jia W Ji L Xiao J. Prevalence of diabetes among men and women in China. N Engl J Med. (2010) 362:1090–101. 10.1056/NEJMoa0908292 20335585

[6] Freund T Campbell SM Geissler S Kunz CU Mahler C Peters-Klimm F. Strategies for reducing potentially avoidable hospitalizations for ambulatory care-sensitive conditions. Ann Fam Med. (2013) 11:363–70. 10.1370/afm.1498 23835823 PMC3704497

[7] Pongiglione B Torbica A Gusmano MK. Inequalities in avoidable hospitalisation in large urban areas: retrospective observational study in the metropolitan area of Milan. BMJ Open. (2020) 10:e042424. 10.1136/bmjopen-2020-042424 33372079 PMC7772299

[8] Agency for Healthcare Research and Quality. Guide to prevention quality indicators:hospital admission for ambulatory care sensitive conditions [EB/OL]. Rockville, MD: AHRQ (2001). Available online at: https://qualityindicators.ahrq.gov/ (accessed December 01, 2008).

[9] Information CIfH. Health Indicators 2013:Definitions, Data Sources and Rationale [EB/OL]. (2013). Available online at: www.cihi.ca

[10] OECD. Health Care Quality and Outcomes (HCQO) 2020-21 Indicator Definitions. (2021). Available online at: https://www.oecd.org/els/health-systems/Definitions-of-Health-Care-Quality-Outcomes.pdf

[11] Ansari Z Carson N Serraglio A Barbetti T Cicuttini F. The Victorian Ambulatory Care Sensitive Conditions study: reducing demand on hospital services in Victoria. Aust Health Rev. (2002) 25:71–7. 10.1071/AH020071 12046157

[12] Fortney JC Steffick DE Burgess JF. Jr., Maciejewski ML, Petersen LA. Are primary care services a substitute or complement for specialty and inpatient services? Health Serv Res. (2005) 40:1422–42. 10.1111/j.1475-6773.2005.00424.x 16174141 PMC1361207

[13] Elek P Molnár T Váradi B. The closer the better: does better access to outpatient care prevent hospitalization? Eur J Health Econ. (2019) 20:801–17. 10.1007/s10198-019-01043-4 30877400 PMC6652173

[14] Hong YR Cardel M Suk R Vaughn IA Deshmukh AA Fisher CL. Teach-back experience and hospitalization risk among patients with ambulatory care sensitive conditions: a matched cohort study. J Gen Intern Med. (2019) 34:2176–84. 10.1007/s11606-019-05135-y 31385206 PMC6816654

[15] Chen HF Popoola T Radhakrishnan K Suzuki S Homan S. Improving diabetic patient transition to home healthcare: leading risk factors for 30-day readmission. Am J Manag Care. (2015) 21:440–50. 26168064

[16] Jia H Chuang HC Wu SS Wang X Chumbler NR. Long-term effect of home telehealth services on preventable hospitalization use. J Rehabil Res Dev. (2009) 46:557–66. 10.1682/JRRD.2008.09.0133 19882490

[17] Iezzi E Lippi Bruni M Ugolini C. The role of GP's compensation schemes in diabetes care: evidence from panel data. J Health Econ. (2014) 34:104–20. 10.1016/j.jhealeco.2014.01.002 24513859

[18] O'Malley AS Reschovsky JD Saiontz-Martinez C. Interspecialty communication supported by health information technology associated with lower hospitalization rates for ambulatory care-sensitive conditions. J Am Board Fam Med. (2015) 28:404–17. 10.3122/jabfm.2015.03.130325 25957373

[19] Cheng-xin J Qin J Hai-long Z Zhen-zhong Z. Analysis on Avoidable Admission of Diabetes in China. Chin Health Econ. (2018) 37:50–3. 10.7664/CHE2018051324479633

[20] Feicheng L Weiyan J Meiping S. Avoidable Hospitalizations for Diabetes in Rural Residents. Chin Gener Pract. (2019) 22:2735–8. 10.12114/j.issn.1007-9572.2019.00.437

[21] Siyuan C Wuping Z Weiyan J. Avoidable Hospitalization for Diabetes and Hypertention in Rural Residents in Central China. Chin Health Qual Manage. (2021) 28:95–7. 10.13912/j.cnki.chqm.2021.28.5.26

[22] Ying H. Study on Potentially Preventable Hospitalization for Diabetic Based on the System of Health Account in Hebei Province. North China: University of Science and Technology (2021).

[23] Kim H Cheng SH. Assessing quality of primary diabetes care in South Korea and Taiwan using avoidable hospitalizations. Health Policy. (2018) 122:1222–31. 10.1016/j.healthpol.2018.09.009 30274936

[24] Youn HM Choi DW Jang SI Park EC. Disparities in diabetes-related avoidable hospitalization among diabetes patients with disability using a nationwide cohort study. Sci Rep. (2022) 12:1794. 10.1038/s41598-022-05557-5 35110602 PMC8810810

[25] Sundararajan V Henderson T Perry C Muggivan A Quan H Ghali WA. New ICD-10 version of the Charlson comorbidity index predicted in-hospital mortality. J Clin Epidemiol. (2004) 57:1288–94. 10.1016/j.jclinepi.2004.03.012 15617955

[26] Kornelius E Huang CN Yang YS Lu YL Peng CH Chiou JY. Diabetes-related avoidable hospitalizations in Taiwan. Prim Care Diabetes. (2014) 8:330–7. 10.1016/j.pcd.2014.02.001 24618275

[27] Weissman JS Gatsonis C Epstein AM. Rates of avoidable hospitalization by insurance status in Massachusetts and Maryland. JAMA. (1992) 268:2388–94. 10.1001/jama.268.17.2388 1404795

[28] Piroozi B Amerzadeh M Safari H Mohamadi-Bolbanabad A Afkhamzadeh A Zarezadeh Y. The burden of preventable hospitalizations before and after implementation of the health transformation plan in a hospital in west of Iran. Prim Health Care Res Dev. (2019) 20:e87. 10.1017/S1463423618000841 32799980 PMC6609975

[29] Shen M He W Yeoh EK Wu Y. The association between an increased reimbursement cap for chronic disease coverage and healthcare utilization in China: an interrupted time series study. Health Policy Plan. (2020) 35:1029–38. 10.1093/heapol/czaa087 32869090

[30] Fan Y. Influence of the improvement of outpatient security level of employees on the utilization of medical services and the expenditure of medical insurance funds: Based on the case analysis of Sanming City, Fujian Province. Chin J Health Policy. (2021) 14:8–13. 10.3969/j.issn.1674-2982.2021.09.002

[31] Feng-mei Z Xiao-juan Z Chun-peng H. The Reform of Outpatient Security System:Policy Effect Analysis of “Replacing Outpatient Service with Hospitalization”-The empirical test based on the sample data of CHIRA. Insurance Stud. (2021) 73–90. 10.13497/j.cnki.is.2021.01.005

[32] Kao YH Lin WT Chen WH Wu SC Tseng TS. Continuity of outpatient care and avoidable hospitalization: a systematic review. Am J Manag Care. (2019) 25:e126–e34. 30986022

[33] Kiadaliri A Englund M. Osteoarthritis and risk of hospitalization for ambulatory care-sensitive conditions: a general population-based cohort study. Rheumatology (Oxford). (2021) 60:4340–7. 10.1093/rheumatology/keab161 33590848 PMC8410004

[34] Wei-yan J Hai F. An empirical analysis on the substitution effect of outpatient services on inpatient services. J Peking Univ (Health Sci). (2015) 47:459–63. 10.3969/j.issn.1671-167X.2015.03.017 26080876

[35] Hu-yang Z Wei-yan J Hai F. Analysis of substitutional effect of outpatient expenditure on inpatient expenditure in hypertensive patients with rural new cooperative medical scheme. J Peking Univ (Health Sci). (2016) 48:472–7. 10.3969/j.issn.1671-167X.2016.03.017 27318910

[36] Dou G Wang Q Ying X. Reducing the medical economic burden of health insurance in China: Achievements and challenges. Biosci Trends. (2018) 12:215–9. 10.5582/bst.2018.01054 29925702

[37] Xin H. Experiences and Lessons from Urban Health Insurance Reform in China. Popul Health Manag. (2016) 19:291–7. 10.1089/pop.2015.0086 26565614

[38] Yang H. Evaluation of the Impact of Basic Medical Insurance on Medical Services in China. Guangxi: Guangxi Medical University (2019).

[39] Zhao C Wang C Shen C Wang Q. China's achievements and challenges in improving health insurance coverage. Drug Discov Ther. (2018) 12:1–6. 10.5582/ddt.2017.01064 29553080

[40] Yu J Qiu Y He Z. Is universal and uniform health insurance better for China? Evidence from the perspective of supply-induced demand. Health Econ Policy Law. (2020) 15:56–71. 10.1017/S1744133118000385 30079859

